# New Challenges in Cancer Control in Japan

**DOI:** 10.2188/jea.JE20120229

**Published:** 2013-03-05

**Authors:** Hiroyuki Noda, Manabu Sumi

**Affiliations:** Cancer Control and Health Promotion Division, Health Service Bureau, Ministry of Health, Labour and Welfare, Tokyo, Japan; 厚生労働省健康局がん対策・健康増進課

## Abstract

2012年6月8日、第二期の「がん対策推進基本計画」の開始が閣議決定された。基本計画の第二期では、がんによる死亡者の減少、全てのがん患者とその家族の苦痛の軽減と療養生活の質の維持向上、がんになっても安心して暮らせる社会の構築を全体目標と設定して、医学的社会的観点から、がん対策の質をさらに改善することとしている。新しい基本計画は、医学的社会的観点に立った国際的ながん対策において、先駆的な挑戦となるであろう。

On June 8, 2012 the Japanese Cabinet decided to launch the second term of the “Basic Plan for Promotion of Cancer Measures”.^1^ The first term was established in 2007 and was scheduled to be revised every 5 years under the terms of the Cancer Control Basic Act.

During the first term of the basic plan (2007–2012), the essential infrastructure for cancer control was successfully improved. The number of designated cancer hospitals increased from 286 to 397, and now 68% of medical districts have such a hospital. All designated cancer hospitals have radiotherapy equipment, an outpatient chemotherapy center, and a center for cancer-related consultation and support. In addition, the number of prefectures with a regional cancer registry increased from 35 to 47 (ie, to all prefectures). Moreover, 30 000 medical doctors engaged in cancer treatment received training in palliative therapy.

In an attempt to add to these improvements, the second term of the basic plan will further improve the quality of cancer control, in both the medical and social contexts, by setting overall goals, namely, decreasing the age-standardized cancer mortality rate, reducing pain and improving the lives of all cancer patients and their relatives, and creating a society in which people with cancer can live comfortably.

To reach these goals, the new basic plan addresses the rapid decrease in the number of surgeons, so as to improve surgical treatment of cancer, and calls for continued improvements in radiotherapy and chemotherapy. The plan also promotes alleviation of pain immediately after a cancer diagnosis and cancer control among infants and people of working age. Cancer survivors often have difficulty finding work and supporting themselves financially after successful cancer treatment: 34% of Japanese workers living with cancer had lost their jobs or had retired. Furthermore, the new basic plan calls for further development of an accurate cancer registry and numerical targets for active and passive smoking and the percentage of the population receiving cancer screening. After the launch of the second term, discussions are already underway regarding enactment of new legislation and the development of more-ambitious cancer control.

By focusing on the social and medical context of cancer, the new basic plan launched by the Japanese government will address challenges in global cancer control amongst cancer patients and cancer survivors.

**Figure.**
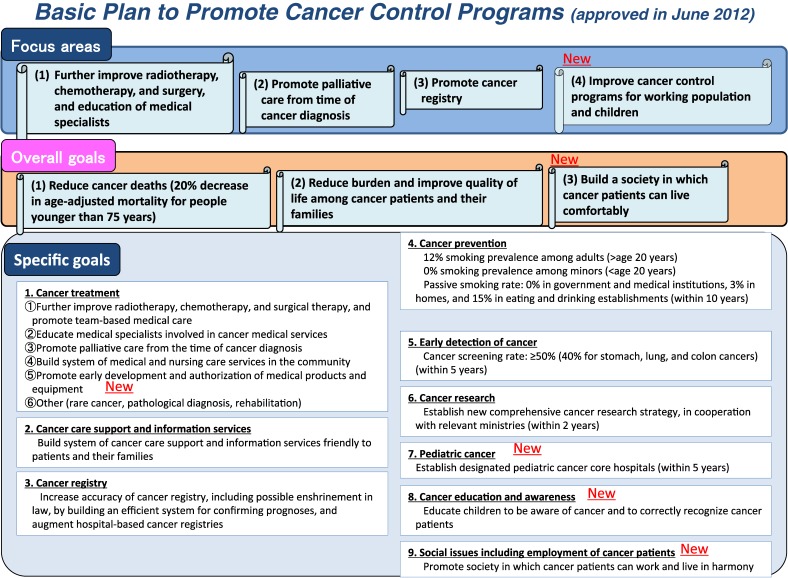


## ONLINE ONLY MATERIALS

Abstract in Japanese.
