# Supplementary material for: New Challenges in Cancer Control in Japan
Source: J Epidemiol. 2013 Mar 5;23(2):153–4. doi: 10.2188/jea.JE20120229 (PMC3700248; doi:10.2188/jea.JE20120229)
Supplement: Abstract in Japanese. [file je-23-153-s001.pdf]

日本のがん対策の新しい挑戦

野田博之、　鷲見学
厚生労働省健康局がん対策・健康増進課

要旨

2012 年 6 月 8 日、第二期の「がん対策推進基本計画」の開始が閣議決定された。基本計画の第二期では、がんによる死亡者の減少、全てのがん患者とその家族の苦痛の軽減と療養生活の質の維持向上、がんになっても安心して暮らせる社会の構築を全体目標と設定して、医学的社会的観点から、がん対策の質をさらに改善することとしている。新しい基本計画は、医学的社会的観点に立った国際的ながん対策において、先駆的な挑戦となるであろう。

キーワード：がん対策、がん対策推進基本計画
